# Intra-Oral Aggressive Fibromatosis: A Systematic Review of Case Reports and Case Series

**DOI:** 10.3390/jcm15041445

**Published:** 2026-02-12

**Authors:** Reem B. Abdelsayed, Mohamed Jaber, Nadin Abouseif, Alaa Mohamed El-Ameen

**Affiliations:** 1 Department of Clinical Sciences, College of Dentistry, Ajman University, Ajman P.O. Box 346, United Arab Emirates; reem.reem@outlook.com; 2 Center of Medical and Bio Allied Health Sciences Research, Ajman University, Ajman P.O. Box 346, United Arab Emirates; alaajabberr@gmail.com; 3 College of Science, United Arab Emirates University, Al Ain P.O. Box 15551, United Arab Emirates

**Keywords:** aggressive fibromatosis, desmoid tumors, desmoid fibromatosis, juvenile aggressive fibromatosis, fibromatosis, oral cavity

## Abstract

**Background/Objectives**: Intra-oral aggressive fibromatosis (IOAF) is a rare, locally invasive soft tissue tumor originating from fibroblasts. Despite its benign histological nature, IOAF exhibits a high recurrence rate and presents significant diagnostic and therapeutic challenges. This systematic review aims to synthesize the available literature on IOAF, focusing on clinical presentation, radiological and histological features, treatment modalities, and recurrence rates. **Methods**: A comprehensive systematic search was conducted, following PRISMA guidelines, across Pubmed, Cochrane, and ScienceDirect for case reports and series detailing IOAF published up to October 2025. This review was registered with PROSPERO (CRD42024586634). Data were extracted on demographics, clinical presentation, radiographic and histological findings, treatment strategies, and follow-up outcomes. Quality of included studies was assessed using the Joanna Briggs Institute (JBI) tools. **Results**: A total of 27 studies were included, encompassing 33 cases. IOAF predominantly affected males (54.5%) with a mean age of 13.15 years. The mandible was the most common site (69.7%). Painless swelling was the most frequent clinical feature (72.7%). Radiological findings were primarily ill-defined radiolucency in 54.5% of the cases. Histopathologically, spindle-shaped fibroblasts and collagen fibers were present in 90% of cases. En bloc resection was the most common treatment (60.6%), followed by excision (27.3%). Recurrence was observed in 12.1% of cases, primarily following conservative treatments. **Conclusions**: IOAF remains a challenging condition due to its locally aggressive nature and potential for recurrence. Surgical resection remains the primary treatment modality, with en bloc resection yielding the lowest recurrence rates. Further research into molecular pathogenesis and targeted therapies is needed to optimize treatment outcomes.

## 1. Introduction

Aggressive fibromatosis (AF), also known as desmoid fibromatosis (DF), is a rare, locally invasive soft tissue tumor originating from fibroblasts, characterized by its tendency for recurrence despite being histologically benign [1]. This disease is often classified into two main categories: extra-oral and intra-oral aggressive fibromatosis, with the latter being a particularly rare and under-recognized manifestation [2,3]. AF is known for its fibroblastic proliferation, which invades the surrounding tissues without metastasis but can cause significant morbidity due to its aggressive local growth [2,4].

Though AF can occur in various locations throughout the body, intra-oral aggressive fibromatosis (IOAF) remains an exceptionally rare entity, with only a few reported cases in the literature [2]. Patients diagnosed with IOAF often present with painless swelling or a firm mass in the oral cavity, most commonly involving the mandible or maxilla [2,3]. The clinical course of IOAF is highly variable, with some lesions demonstrating rapid growth while others remain stable or regress spontaneously.

The lack of a clear pathognomonic feature for diagnosis and its often non-specific clinical presentation overlap with a wide spectrum of benign fibroblastic, myofibroblastic, odontogenic, and low-grade malignant lesions of the oral cavity, often necessitating careful clinicoradiologic–histopathologic correlation for early detection and accurate diagnosis [1,4]. Consequently, IOAF is not a diagnosis of exclusion but rather one that requires integration of clinical behavior, imaging findings, and characteristic histopathological features.

Histologically, AF is characterized by spindle-shaped fibroblasts arranged in a collagenous matrix, and the mitotic figures are generally low but may be present, and their presence without cytologic atypia helps distinguish IOAF from malignant neoplasms [4]. The tumors may exhibit a range of radiological features, from well-defined lesions with mild bone involvement to more aggressive patterns with extensive osseous destruction. Treatment typically involves surgical resection, although the recurrence rate remains high, even with complete excision. Adjuvant therapies such as radiotherapy, chemotherapy, or targeted therapies have been used in selected cases, particularly when surgery is not feasible or when the tumor exhibits aggressive behavior [1,5].

Despite the available data, significant gaps remain in understanding the precise management strategies and clinical behaviors of intra-oral aggressive fibromatosis, particularly in relation to its long-term outcomes and recurrence rates. This systematic review aims to provide a comprehensive summary of the existing literature on intra-oral AF, focusing on its clinical features, radiological findings, histopathological characteristics, treatment modalities, and recurrence rates, in order to inform future clinical practice and research on this rare disease.

## 2. Materials and Methods

### 2.1. Protocol and Registration

This systematic review was reported in accordance with the guidelines of the Preferred Reporting Items for Systematic Reviews and Meta-Analyses (PRISMA) checklist and was prospectively registered with PROSPERO [ID: CRD42024586634] [6].

### 2.2. Search Strategy and Resources

A comprehensive and structured search strategy was applied across three major electronic databases: PubMed, Cochrane Library, and ScienceDirect. The search encompassed all available literature from database inception to the final search date (October 2025). Search terms were selected to maximize sensitivity and included controlled vocabulary and free-text expressions such as “Aggressive Fibromatosis,” “Desmoid Fibromatosis,” “Juvenile Aggressive Fibromatosis,” and “Fibromatosis.” Boolean operators, field-specific tags, and Advanced Search Builder functions were used to refine retrieval. Search filters were applied to limit results to human studies and articles published in the English language.

Records were reviewed by title and abstract by two independent reviewers (R.A. and N.A.). Chosen studies were assessed for eligibility by reading the full text post-retrieval. To ensure methodological rigor, a continuously updated exclusion log was maintained, documenting the reason for excluding each non-eligible study. When the reviewers had differing views, they resolved the issue through discussion, and a third senior reviewer (M.J.) was consulted if consensus could not be reached.

### 2.3. Eligibility

The studies included in this review for synthesis conformed to all predefined criteria established according to the PICOS framework, encompassing population, intervention, comparison, outcomes, and study design (Table 1). Only cases diagnosed as intra-oral aggressive fibromatosis based on characteristic histopathological findings in conjunction with clinically or radiologically documented local invasiveness were included.

### 2.4. Data Extraction

Before commencing data synthesis from the final list of included articles, all extracted variables were harmonized and standardized to ensure consistency in terminology, reporting units, and descriptive parameters across the included case reports and case series. This step facilitated accurate comparison and integrative analysis of clinical, diagnostic, and therapeutic data. Data extracted from the included studies encompassed the following variables: author(s) and year of publication, sample characteristics (e.g., age, gender, location), clinical features (e.g., symptoms, duration), radiological findings, histopathological characteristics, type of intervention, follow-up details, and recurrence outcomes.

### 2.5. Risk of Bias Assessment

The critical appraisal of case reports was assessed according to the Joanna Briggs Institute critical appraisal tools for JBI Systematic Reviews issued by the Faculty of Health and Medical Sciences at the University of Adelaide, South Australia [7]. The appraisal was independently performed by two reviewers and is presented (Table S2). Each domain of the JBI instrument was evaluated, and inconsistencies were discussed until consensus was reached. This approach ensured a standardized and transparent assessment of internal validity.

## 3. Results

**Figure 1:**
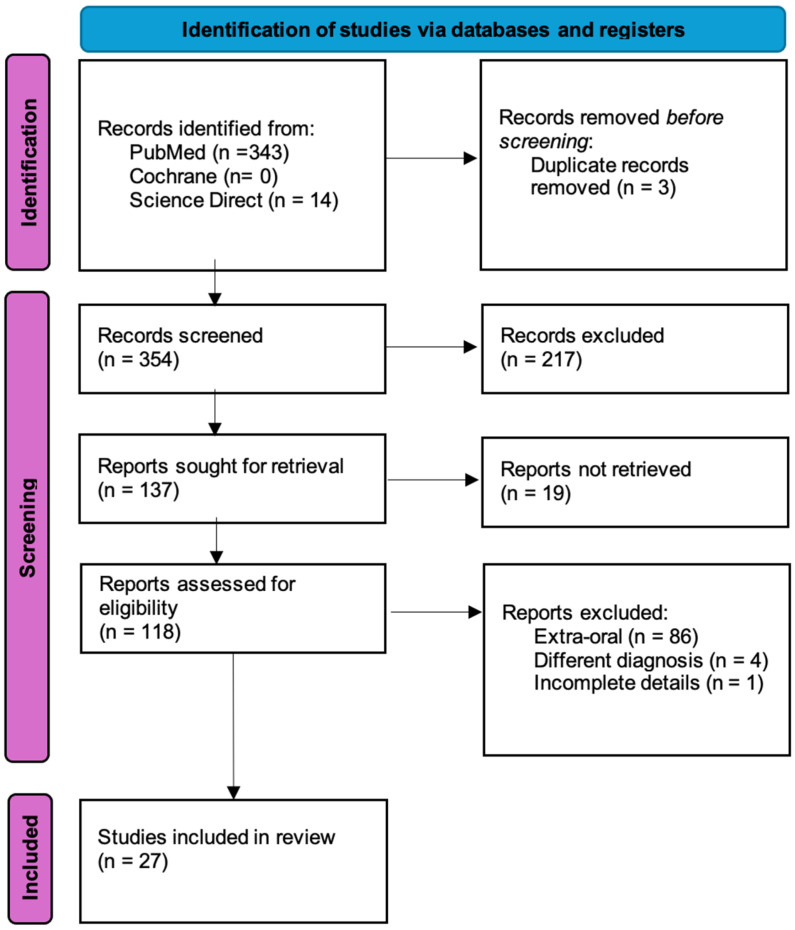
PRISMA flow diagram.

This systematic review synthesized data from 27 studies, selected from an initial pool of 118 studies assessed for eligibility, which included a total of 33 cases (Figure 1). The data is summarized in Table 2 and Table 3. Details of histological findings for each case are presented (Table S1).

Of the 27 articles, 18 scored high quality and nine scored moderate quality. The single-case design of most reports limits the robustness of the evidence, warranting cautious interpretation of the results (Table S2).

To explore potential demographic and anatomical trends, descriptive subgroup analyses were performed based on patient age (pediatric ≤ 18 years vs. adult > 18 years) and lesion location (mandible, maxilla, tongue). Given the rarity of IOAF and the case-based nature of available evidence, analyses were descriptive rather than inferential. Diagnosis relies on clinical–radiological–histopathological correlation.

The reported age range of patients varied from 2 weeks to 50 years, with a mean age of approximately 13.15 years. The gender distribution indicated a higher prevalence in males (54.5%) compared to females, with a ratio of 1.2:1. The majority of cases occurred in the pediatric population. Specifically, 26 of 33 cases (78.8%) were diagnosed in patients aged 18 years or younger, while only seven cases (21.2%) involved adults. Notably, 18 cases (54.5%) occurred in children younger than 10 years, underscoring a strong predilection for early childhood and adolescence.

Anatomical distribution analysis demonstrated a marked predilection for the mandible, which accounted for 23 of 33 cases (69.7%). Within the mandible, lesions most frequently involved the posterior regions, including the ramus, angle, and posterior body. Specifically, mandibular ramus and angle involvement—either isolated or combined with adjacent regions—were reported in 14 cases (42.4%). Lesions confined to the mandibular body or mid-mandible were observed in six cases (18.2%), while anterior mandibular involvement accounted for three cases (9.1%).

Maxillary involvement was less common, reported in six cases (18.2%). These lesions primarily affected the posterior maxilla and maxillary sinus, often extending into adjacent alveolar bone or sinus walls. Isolated palatal involvement was rare and observed in only two cases.

Tongue involvement was identified in four cases (12.1%), predominantly affecting the anterior or base of the tongue. These lesions were typically soft tissue-dominant and demonstrated limited or absent osseous involvement compared to mandibular and maxillary cases.

Clinically, the most frequent presentation was painless swelling, observed in 72.7% of cases, while pain was less commonly reported (7/33 cases).

Osseous destruction was a prominent feature, identified in 54.5% of cases, typically affecting the cortical plates of the mandible. Among these, 27.3% exhibited full-thickness cortical involvement. This presented radiologically as ill-defined or irregular radiolucencies. Histopathological analysis across the 33 reported cases demonstrated a highly consistent pattern characteristic of aggressive fibromatosis. Spindle-shaped fibroblasts or myofibroblasts were identified in 30 of 33 cases (90.9%), making this the single most defining feature. These cells were typically arranged in interlacing fascicles, whorled bundles, or wavy fiber patterns, observed in 21 cases (63.6%). Collagen deposition was prominent, with collagen-rich stroma reported in 24 cases (72.7%), while hyalinization was noted in six cases (18.2%). Myxoid stromal change, a recognized but less frequent variant, was present in five cases (15.2%). Mitotic activity was generally low; however, mitotic figures were documented in four cases (12.1%), all without cytologic atypia. Giant cell components (osteoclast-like or multinucleated) were noted in two cases (6.1%).

Regarding management, en bloc resection emerged as the predominant treatment approach, employed in 60.6% of cases. Local excision was performed in 27.3%, while combined modalities (including adjunctive chemotherapy or radiotherapy) were utilized in 12.1% of cases. Follow-up periods ranged from 1 to 10 years, with a mean duration of approximately 3 years. Recurrence occurred in four cases (accounting for 12.1%) (Table 3). Recurrence analysis stratified by age demonstrated similar recurrence frequencies between pediatric and adult patients. Among the 26 pediatric cases (≤18 years), recurrence was reported in three cases (11.5%). In the adult group (>18 years; n = 7), recurrence occurred in one case (14.3%). Overall, four of 33 cases (12.1%) experienced recurrence during the reported follow-up period.

## 4. Discussion

Intra-oral aggressive fibromatosis (IOAF) represents an exceptionally uncommon manifestation of desmoid-type fibromatosis, and its rarity continues to pose significant diagnostic and therapeutic challenges. This systematic review offers clinically meaningful insights into presentation patterns, diagnostic characteristics, and treatment outcomes that can guide patient evaluation and surgical planning.

A notable demographic feature observed across the reviewed cases is the marked predilection for younger individuals, with a mean age of 13.15 years and nearly half of patients younger than 10 years. This aligns with the known bimodal age distribution of desmoid tumors but underscores that IOAF should remain on the differential diagnosis for pediatric mandibular and maxillary masses [33]. The slight male predominance in this review contrasts with the female predominance often reported in extra-oral desmoid tumors [34], suggesting that IOAF may biologically or etiologically differ from its extra-oral counterparts. Given the non-specific presentation and overlap with more common entities, IOAF is likely under-recognized, especially in settings without access to advanced imaging or specialist pathology review. This further underscores the need for increased awareness and multidisciplinary evaluation.

Clinically, IOAF most frequently presents as a slowly enlarging, painless swelling, an inherently non-specific sign that overlaps with a range of benign odontogenic, myofibroblastic, and vascular lesions [35,36,37,38]. The mandible, particularly the ramus and posterior body, accounted for 71.9% of cases, making this region a key anatomical site where clinicians should consider IOAF when evaluating progressive intra-oral masses in children. Pain, ulceration, or functional disturbances such as reduced mouth opening were less frequent but tended to occur in rapidly enlarging or more invasive lesions. The predominance of mandibular involvement—particularly the ramus and posterior body—suggests a potential relationship between IOAF development and areas of active bone remodeling and masticatory stress during growth. This anatomical predilection mirrors patterns observed in pediatric desmoid-type fibromatosis of the head and neck, where deep fascial planes and periosteal interfaces may facilitate infiltrative growth. In contrast, maxillary and lingual lesions were less frequent and tended to present with more localized soft tissue involvement, potentially influencing surgical accessibility and recurrence risk.

Radiographically, approximately 54.5% of cases demonstrated osseous destruction presenting as ill-defined radiolucency in most cases. These findings highlight the importance of early cross-sectional imaging, as IOAF may initially mimic benign fibro-osseous lesions but can progress to deeper invasion, influencing the feasibility of conservative excision. The variability of radiologic appearance—from ill-defined radiolucencies to multiloculated lesions—reinforces the necessity of correlating imaging findings with histopathology.

Histologically, the consistent presence of spindle-shaped fibroblasts and a collagenous matrix across cases supports the classic diagnostic criteria of aggressive fibromatosis [4]. However, the spectrum ranging from myxoid to densely collagenized lesions and the occasional presence of mitotic figures emphasize the need for careful exclusion of low-grade sarcomas. Given the diagnostic overlap with myofibromas, nodular fasciitis, and low-grade fibrosarcomas, integration of clinical behavior, imaging, and close margin assessment remains essential [39].

From a management standpoint, our findings reinforce that complete surgical excision with clear margins remains the most reliable strategy for preventing recurrence [5]. En bloc resections demonstrated the lowest recurrence rate, whereas conservative excision or curettage showed higher failure rates, mirroring patterns reported in head and neck desmoid tumors more broadly. Determinants of therapeutic decision-making include factors such as lesion size, extent of invasion, and patient age, which provides insights into indications for multimodal therapy and balancing radical resection with growth preservation in pediatric populations. Importantly, recurrence was identified in 12.1% of cases, with most recurrences occurring after incomplete or conservative surgical approaches. While adjuvant treatments such as radiotherapy or chemotherapy were employed in selected cases—typically for unresectable or recurrent tumors—the heterogeneity of indications and outcomes limits firm conclusions. Thus, there is a need for future studies to more precisely document treatment details, including margin status, to enable finer-grained comparative effectiveness analyses. In extra-oral desmoid tumors, the use of radiotherapy and chemotherapy as adjunct treatment along with surgery has been found to be effective in recurrent cases [40,41]. Nonetheless, these modalities remain valuable adjuncts when surgical morbidity is prohibitive or when achieving tumor-free margins is not possible. Further research is needed to evaluate the efficacy of these therapies, particularly in cases where complete surgical resection is not feasible [5]. Emerging paradigms from extra-oral desmoid tumor management (such as active surveillance and systemic targeted therapies) suggest that similar approaches could be considered for selected IOAF cases, especially where surgery would cause significant functional or cosmetic morbidity; thus, future research to evaluate these alternatives in the oral context is warranted.

Clinically, these data suggest that early identification and timely referral are crucial for achieving resectability while minimizing functional and developmental compromise, particularly in pediatric patients. The aggressive local behavior of IOAF, combined with its benign histology, places clinicians in the important position of balancing oncologic control with preservation of growth potential, occlusion, mandibular integrity, and facial symmetry. Multidisciplinary management—incorporating oral and maxillofacial surgery, radiology, pathology, and pediatric oncology—is essential for optimizing outcomes.

### Recommendations for Clinical Practice

Based on the current, low-certainty evidence, the following framework is proposed to guide clinical management:

1. Diagnostic Pathway
  - MRI is the preferred initial study for assessing marrow and soft-tissue involvement. Non-contrast CT should complement MRI to detail bony architecture and matrix patterns. Core needle biopsy is recommended for histopathological confirmation, targeting viable lesional tissue.
2. Surgical and Adjuvant Management
  - The main goal is complete excision with local control. A 1–2 cm macroscopic margin is recommended where feasible. In critical anatomic sites, functional preservation may necessitate closer margins.
  - The role of radiotherapy or systemic agents (e.g., tyrosine kinase inhibitors) is not yet defined. Consideration may be individualized for cases with positive margins, large tumor size (>5 cm), or unresectable recurrence.
3. Given the risk of late recurrence, structured follow-up with a clinical exam and MRI every 6–12 months for 3–5 years, then annually, is advised.

## 5. Study Limitations

The findings of this review must be interpreted within the limitations inherent to rare disease literature, notably small sample sizes, retrospective case-based evidence, and variability in follow-up duration. Inconsistent reporting of margin status and adjuvant therapy indications further complicates comparative analysis. Furthermore, many included studies (retrospective case reports/series) lacked detailed documentation of imaging modalities (CT/MRI), surgical margin assessment, or precise recurrence timing. Given the case-based nature of the evidence and small sample sizes, quantitative heterogeneity testing was not performed; this may have a serious impact on the robustness of the findings.

## 6. Recommendations

To address the current gaps in knowledge and optimize patient care, future efforts should be concentrated on three interrelated fronts:

1. Initiating large, multicenter prospective cohort studies with standardized data collection protocols is paramount. This will provide the high-quality evidence needed to define natural history, identify true risk factors, and evaluate long-term outcomes.
2. Research must move beyond descriptive histology to explore underlying molecular mechanisms, such as the role of *CTNNB1* mutations and other dysregulated pathways. A critical goal is to determine how these mechanisms correlate with clinical behavior and treatment response, paving the way for personalized therapeutic strategies.
3. There is an urgent need to develop and adopt comprehensive diagnostic and management guidelines to harmonize clinical care. Concurrently, establishing standardized reporting frameworks for key variables, including detailed lesion dimensions, quantitative imaging features, and validated histopathological classifications (e.g., collagen patterns), and follow-up periods to better understand the long-term outcomes of this rare condition, as well as clarify the role of non-surgical therapies and develop evidence-based surveillance protocols, is essential. This standardization will enable reliable aggregation and systematic correlation analysis across studies.

## 7. Conclusions

In conclusion, IOAF is a rare but clinically significant entity with the potential for destructive local invasion. Painless swelling of the mandible in children should raise suspicion, prompting early imaging and biopsy. Complete surgical excision remains the most effective treatment, with recurrence risk concentrated in cases managed conservatively. Improving awareness of IOAF’s clinical behavior and advancing structured reporting will be key to refining management strategies and improving long-term patient outcomes. Further research is needed to explore the role of adjuvant therapies and to establish standardized treatment protocols for this rare disease.

## Figures and Tables

**Table 1:** PICOS inclusion and exclusion criteria.

Parameter	Inclusion	Exclusion
Population	Patients of all ages diagnosed with intra-oral aggressive fibromatosis	Patients NOT diagnosed with aggressive fibromatosis or presented with extra-oral AF with no intra-oral features
Intervention	Treatment of intra-oral features of aggressive fibromatosis (surgical/therapeutic. etc.)	Treatment of extra-oral-only features
Comparison	N/A	N/A
Outcome	Clinical features Radiographic and histopathological findings Treatment, outcome and enough follow-up	Incomplete demographical, clinical, histopathological and follow-up details
Study design	Case reports and case series Full-text availability Any interpreted language	Review articles, editorials, animal studies and conference abstracts Duplicates that may introduce bias

**Table 2:** Characteristics of included studies.

Author	Age/Sex	Location	Symptoms	Duration	Radiological Appearance	Bony Involvement/Extension	Treatment	Recurrence	Follow Up
Shapiro & Goffin 1968 [8]	16 years F	Ramus of mandible	Pain, swelling, reduced mouth opening	1 month	Osseous destruction	Total bone thickness	Total hemimandibulectomy (En bloc resection)	0	6 months
Larson & Bjorlin 1976 [9]	10 years F	Lingual of mandible	Bleeding rapidly progressing mass	1 month	Osseous destruction	Cortex	Total excision, tooth extraction	0	2 years
	11 years F	Lingual of mandible	Ulcerated mass	3 weeks	Osseous destruction	Cortex	Total excision, tooth extraction	0	2 years
Schwartz et al. 1979 [10]	16 years M	Tongue	Hard mass, discomfort, otalgia	6 years	N/A	N/A *	Lateral pharyngotomy and resection	0	2 years
Melrose & Abrams 1980 [11]	3 years F	Ramus and angle of mandible	Rocky hard swelling, discomfort	1 month	Ill-defined radiolucency	Cortex	Curettage, actinomycin, radiation	1	9 years
	5 years F	Palatal to maxilla	Red ulcerated swelling	1 month	Ill-defined radiolucency with root resorption	Alveolar ridge	Alveolectomy and excision	0	4 years
	3 years M	Posterior mandible	Swelling	6 months	Cortex destruction, radiolucency	Cortex	En bloc resection	0	5 years
Rodu et al. 1981 [12]	2 years M	Posterior mandible	Painless swelling	2 weeks	N/A	Cortex	En bloc resection	0	6 years
	22 years M	Posterior mandible	Painless swelling	1 month	N/A	Cortex	En bloc resection	0	2.5 years
	11 years F	Posterior mandible	Painless swelling	2 months	N/A	Cortex	En bloc resection	0	2 years
	16 years F	Mid-mandible	Painless swelling	2–3 months	N/A	Cortex	En bloc resection	0	3 years
Petri et al. 1982 [13]	42 years M	Buccal to mandibular alveolus	Painful mass	N/A	N/A	Alveolar ridge	En bloc resection	0	5 years
Slootweg & Muller 1984 [14]	2 weeks M	Buccal to mandibular alveolus	Painless swelling	Born with	Osseous destruction, teeth displacement.	Alveolar ridge	Total excision	0	10 years
Geist et al. 1985 [15]	2 years M	Body and angle of mandible	Painless firm swelling	3 months	Moderately well-defined multiloculated lesion	Total bone thickness	En bloc resection	0	1 year
Zachariades et al. 1988 [16]	6 years M	Anterior mandible	Hard mass	1 month	N/A	Alveolar ridge	En bloc resection	0	5 years
Shah et al. 1988 [17]	6 months M	Tongue Base	Stridor, cough, respiratory distress and firm mass	5 months	N/A	N/A	Labial mandibuloglossotomy and resection	0	1 year
Chen et al. 1989 [18]	9 years M	Anterior third of tongue	Painless sore, difficulty swallowing	1 month	Well-defined homogenous mass	N/A	Chemotherapy, en bloc resection, radiotherapy	0	1.5 year
Donohue et al. 1990 [19]	14 years F	Hard and soft palate	Painless firm swelling	few months	N/A	Alveolar process	En bloc resection	0	3 years
Steuer et al. 1995 [20]	2 years M	Retromolar, angle, ramus and submandibular	Painless swelling, snoring and drooling	4 months	“Onion skinning”, osseous destruction	Total bone thickness	En bloc resection	0	3 months
De Santis et al. 1998 [21]	16 months F	Angle of mandible	Painless mass	1 month	N/A	No involvement	Total excision	0	4 years
Akama et al. 2002 [22]	38 years F	Alveolar ridge to ramus of mandible	Painless firm swelling, mobile molars	1 year	Osseous destruction, teeth displacement.	Cortex	En bloc resection	1	4 months
Watzinger et al. 2005 [23]	7 years F	Body and ramus of mandible	Painless mass	3 years	Cortex destruction	Total bone thickness	Segmental resection	0	1.5 years
Seper et al. 2005 [24]	4 years M	Angle and body of mandible	Painless hard swelling	1 month	Ill-defined radiolucency	Total bone thickness, unresectable	Excision, tumor debulking, chemotherapy	1	1 year
De Riu et al. 2006 [25]	2 years F	Body of mandible	Painless hard swelling	N/A	Radiopaque lesion with ill-defined margins	Cortex	En bloc resection	0	1.5 years
Suresh & Ali 2008 [26]	22 years F	Tongue	Painless swelling	4 months	N/A	N/A	Surgical excision	0	10 years
Rao et al. 2011 [3]	50 years F	Maxilla + maxillary sinus	Non-tender swelling	2 months	Radiolucent-radiopaque soft tissue lesion	Total bone thickness	Partial maxillectomy and excision + radiotherapy	0	4 years
Zaki et al. 2012 [27]	2 years M	Mandible, floor of the mouth	Painless swelling	7 months	Osteolytic radiolucency	Total bone thickness	Partial mandibular resection	0	2 years
Kawamata et al. 2013 [28]	40 years M	Maxilla + maxillary sinus	Hard swelling with redness and dull pain	3 years	Eroded posterior walls of sinus and alveolar bone	Total bone thickness	Caldwell-Luc excision	0	2.5 years
Ivanov et al. 2013 [29]	17 years F	Body of mandible	Painful, mobile premolar	6 months	Radiolucency, premolar root resorption	Total bone thickness	Complete resection, tooth extraction	0	1 year
Nair et al. 2017 [2]	5 years M	Alveolar ridge of posterior mandible	Painless swelling, with mild bleeding	2 months	Well-defined radiolucency with discontinuous sclerotic margins	N/A	Total excision of mass and developing tooth bud	0	3 months
Sivanandham et al. 2022 [30]	4 years M	Angle, ramus and lower border of mandible	Pain and swelling, reduced mouth opening	2 months	Irregular radiolucency with ill-defined margins	Cortex, involving condylar process	Segmental mandibulectomy and excision	0	3 months
Sahni et al. 2024 [31]	36 years M	Hard palate	Gradual painless swelling	6 months	Heterogeneous mass	N/A	Total maxillectomy and resection	0	3 months
Yang et al. 2025 [32]	15 years M	Left posterior mandible	Pain, swelling, intra-oral mass formation, tongue displacement and left lower lip numbness	2 months	Local bone destruction	Cortex	En bloc resection	1	14 months

* NA = not reported.

**Table 3:** Summary of results.

	Total	Average
**Demographics**		
Female	15/33	45.5%
Male	18/33	54.5%
Adult (>18 years)	7/33	21.2%
Pediatric (<18 years)	26/33	78.8%
**Location**		
Maxilla	6/33	18.2%
Tongue	4/33	12.1%
Mandible	23/33	69.7%
**Symptoms**		
Painless swelling/mass	24/33	72.7%
Painful swelling/mass, discomfort	7/33	21.2%
Ulcerated mass/swelling	2/33	6.1%
**Radiographic Features**		
Radiolucency/osseous destruction	18/33	54.5%
Homogeneous/heterogeneous/radiopaque mass	3/33	9.1%
Radiolucent–radiopaque lesion	1/33	3.0%
**Bone Involvement**		
Total bone thickness	9/33	27.3%
Cortex	12/33	36.4%
Alveolar bone	5/33	15.2%
No involvement	1/33	3.0%
**Treatment Modalities**		
En bloc resection	20/33	60.6%
Excision	9/33	27.3%
Combination with chemotherapy or radiotherapy	4/33	12.1%
**Follow-up**	-	3 years
**Recurrence**	4/33	12.1%

## Data Availability

The original contributions presented in this study are included in this article. Further inquiries can be directed to the corresponding authors.

## Supplementary Materials

The following supporting information can be downloaded at: https://www.mdpi.com/article/10.3390/jcm15041445/s1. Table S1. Histopathological findings. Table S2. JBI quality assessment of case reports. Table S3. PRISMA checklist.
