# Supplementary material for: Intra-Oral Aggressive Fibromatosis: A Systematic Review of Case Reports and Case Series
Source: J Clin Med. 2026 Feb 12;15(4):1445. doi: 10.3390/jcm15041445 (PMC12942575; doi:10.3390/jcm15041445)
Supplement: Supplementary file 1 [file jcm-15-01445-s001.zip › Table S2. JBI quality assessment of case reports.pdf]

Table S2. JBI quality assessment of case reports.

Author	Were Patient's Demographic Characteristics Clearly Described?	Was the Patient's History Clearly Described and Presented as a Timeline?	Was the Current Clinical Condition of the Patient on Presentation Clearly Described?	Were Diagnostic Tests or Assessment Methods and the Results Clearly Described?	Was the Intervention(s) or Treatment Procedure(s) Clearly Described?	Was the Post-Intervention Clinical Condition Clearly Described?	Were Adverse Events (Harms) or Unanticipated Events Identified and Described?	Does the Case Report Provide Takeaway Lessons?	Overall appraisal:
Shapiro & Goffin[8]	YES	NO	YES	YES	YES	YES	N/A	YES	**6**
Larson & Bjorlin[9]	YES	NO	YES	YES	YES	YES	N/A	YES	**6**
Schwartz et al.[10]	YES	YES	YES	YES	YES	YES	YES	YES	**8**
Melrose & Abrams[11]	YES	NO	YES	YES	YES	YES	YES	YES	**7**
Rodu et al.[12]	YES	YES	YES	YES	NO	NO	N/A	YES	**5**
Petri et al.[13]	YES	YES	YES	YES	YES	YES	YES	YES	**8**
Pieter et al.[14]	YES	YES	YES	YES	YES	YES	N/A	YES	**7**
Geist et al[15]	YES	YES	YES	YES	YES	YES	YES	YES	**8**
Zachariades et al[16]	YES	YES	YES	YES	YES	YES	N/A	YES	**7**
Shah et al.[17]	YES	YES	YES	YES	YES	YES	N/A	YES	**7**
Chen et al.[18]	YES	NO	YES	YES	YES	YES	YES	YES	**7**
Donohue et al.[19]	YES	NO	YES	YES	YES	YES	YES	YES	**7**
Steuer et al.[20]	YES	YES	YES	YES	YES	NO	N/A	YES	**6**
De Santis et al.[21]	YES	YES	YES	YES	YES	YES	N/A	YES	**7**
Akama et al.[22]	YES	YES	YES	YES	YES	YES	YES	YES	**8**
Watzinger et al.[23]	YES	NO	YES	YES	YES	YES	N/A	YES	**6**
Seper et al[24]	YES	YES	YES	YES	YES	YES	YES	YES	**8**
De Riu et al.[25]	YES	YES	YES	YES	YES	YES	YES	YES	**8**
Suresh & Ali[26]	YES	YES	YES	YES	YES	YES	N/A	YES	**7**
Zaki et al.[27]	YES	NO	YES	YES	YES	YES	N/A	YES	**6**
Rao et al.[3]	YES	YES	YES	YES	YES	YES	YES	YES	**8**
