# Supplementary material for: Intra-Oral Aggressive Fibromatosis: A Systematic Review of Case Reports and Case Series
Source: J Clin Med. 2026 Feb 12;15(4):1445. doi: 10.3390/jcm15041445 (PMC12942575; doi:10.3390/jcm15041445)
Supplement: Supplementary file 1 [file jcm-15-01445-s001.zip › Table S1. Histopathological findings.pdf]

Table S1. Histopathological findings

Location	Histopathology
Ramus of mandible	spindle cells, myxoid change, hyalinization
Lingual of mandible	spindle shaped cells, whirl like bundles, mitotic figures
Lingual of mandible	highly collagenized area, cord-like interlacing fasculi
Tongue	irregularly thick SSE, pegs superficially
Ramus and angle of mandible	fibroblastic proliferation, wavy fibrillar pattern
Palatal to maxilla	large capillaries lined with plump endothelial cells
Posterior mandible	nonencapsulated elongated fibroblasts with collagen fibers
Posterior mandible	Spindle shaped cells in fibrous connective tissue
Posterior mandible	Spindle shaped cells in fibrous connective tissue
Posterior mandible	Spindle shaped cells in fibrous connective tissue
Mid-mandible	Spindle shaped cells in fibrous connective tissue
Buccal to mandibular alveolus	stellate and spindle-shaped fibroblasts, collagen fibers, "checkerboard" appearance
Buccal to mandibular alveolus	elongated fibroblasts, intracytoplasmic longitudinal fibrils.
Body and angle of mandible	elongated fibroblasts, loosely arranged myxoid tissue, collagen fibers, stellate nuclei cells.
Anterior maxilla	bundles of highly cellular connective tissue surrounded by fibro-fatty tissues
Tongue Base	cellular fibroblasts, hyperchromatic nuclei, rare mitosis, stromal component with collagen fibers and loose myxomatous tissue
Anterior third of tongue	Fibroblasts proliferation, fibrosis and collagen.
Hard & soft palate	highly cellular connective tissue, multidirectional bundles of fibers
Retromolar, angle, ramus and submandibular	Fibroblasts, no atypical aspects, low mitotic index
Angle of mandible	Fibroblasts "cigar shaped cells", low mitotic index
Alveolar ridge to ramus of mandible	spindle shaped with mitotic figures
Body and ramus of mandible	Spindle shaped cells, collagen fibers and fibroblasts.
Angle and body of mandible	mesenchymal tumor with spindle-shaped tumor cells, fibroblasts, collagen fibers
Body of mandible	juvenile aggressive fibromatosis
Mandible, floor of the mouth	myofibroblastic spindle cells with an active nucleolated nucleus with rich collagenous stroma.
Body of mandible	Spindle-like cells, whirl like bundles, mitotic figures, giant cell osteoclasts
Alveolar ridge of posterior mandible	Spindle cells in 1ascicles, parallel blood vessels
Tongue	Fibrous tissue showing glossy, eosinophilic, hyalinized areas with a perivascular distribution. Condensed fibrous tissue showed moderate cellularity with focal areas of mild inflammatory reaction. Slit-like vascular spaces were seen scattered throughout the connective tissue
Maxilla & maxillary sinus	Interlacing bundles of elongated spindle-shaped cells, showing moderate anisonucleosis, with capillary channels in between. Focal areas of hyalinization with peripheral arrangement of cells were observed. Few foci of giant cells were also seen
Maxilla & maxillary sinus	Heavily collagenized stroma with spindle cells with bland nuclei and elongated vessels
Angle, ramus and lower border of mandible	Numerous spindle shaped fibroblasts like cells with a bland nucleus and a prominent nucleoli.
Hard palate	Fibrinous material, areas of necrosis and proliferated spindle cells.
